# Short- and Long-Term Effects of Undernutrition During Adolescence on Oxidative Status and Glucose Homeostasis in Male and Female Rats

**DOI:** 10.3390/biology14101352

**Published:** 2025-10-02

**Authors:** Joskame Saint Paul, Antônio José Rocha Ribeiro, Ana Caroline Schoenberger Kipper, Mariele de Oliveira Souza, Thiara Chaves dos Santos, Karoline Paiva da Silva, Aline Milena Dantas Rodrigues, Manoela Fontenele Antunes, Isabelle Zanata Fabiane, Ana Júlia Lopes Braga Ferneda, Valéria Dornelles Gindri Sinhorin, Renata de Azevedo Melo Luvizotto, Júlio Cezar de Oliveira

**Affiliations:** 1 Research Group on Perinatal Programming of Metabolic Diseases: DOHaD Paradigm, Federal University of Mato Grosso, University Campus of Sinop, Sinop 78557-267, MT, Brazil; joskameclerge@gmail.com (J.S.P.); mariele.souza@sou.ufmt.br (M.d.O.S.); thiara.enf@gmail.com (T.C.d.S.);; 2 Laboratory of Metabolic and Cardiovascular Diseases, Health Education and Research Center (NUPADS), Institute of Health Sciences, Federal University of Mato Grosso, University Campus of Sinop, Sinop 78557-267, MT, Brazilrenata.nascimento@ufmt.br (R.d.A.M.L.); 3 Integrated Laboratory of Chemical Research (LiPEQ), Institute of Exact and Earth Sciences, Federal University of Mato Grosso, University Campus of Sinop, Sinop 78557-267, MT, Brazil

**Keywords:** metabolic programming, undernutrition, puberty, oxidative stress

## Abstract

Malnutrition during adolescence can cause metabolic diseases later in life. This study examined the short- and long-term effects of undernutrition during adolescence on body composition, glucose homeostasis and redox balance. Male (*n* = 32) and female (*n* = 32) Wistar rats were fed a rodent chow reduced by 50% (FR50) of the amount consumed by control rats (CONT) from 30 to 60 days and then fed ad libitum until 120 days of age. Half of the rats were euthanized at 60 and the other half at 120 days old. At 60 and 120 days old, glucose and insulin tolerance test; skeletal muscle, visceral fat, liver and interscapular brown adipose tissue (iBAT) weights; and oxidative stress marker levels in the liver and iBAT were evaluated. The FR50 male (FR50-M) and female (FR50-F) rats exhibited a lean phenotype and high insulin sensitivity at 60 days of age (*p* < 0.05), but at 120 days of age, they exhibited an obese phenotype with high insulin sensitivity (*p* < 0.05). An increase in liver GSH was observed as only a short-term effect (*p* < 0.05). At 120 days of age, only male rats displayed increased iBAT GSH levels (*p* < 0.05) and reduced CAT activity (*p* < 0.01). In summary, undernutrition during adolescence affects body composition, glucose homeostasis and redox status equally in males and females but causes long-term impairment of the redox status of iBAT only in male rats.

## Simple Summary

Undernutrition, particularly in critical stages of life development, such as adolescence, can lead to lifelong energy and glucose dyshomeostasis. When dietary protein is inadequate during adolescence, it has long-term consequences in male rats, leading to an obesity phenotype associated with insulin resistance and hypothalamus-pituitary-gonadal axis and testosterone disruption. Accordingly, this study was designed to evaluate both the immediate (short-term) and extended (long-term) effects of undernutrition resulting from food deprivation (by restricting caloric intake to 50% of that consumed by the control group of rats) on body composition, glucose–insulin homeostasis and redox balance markers in metabolic (liver) and thermogenic interscapular brown adipose (iBAT) tissues in both male and female rats. The body of data in the present article provides novel information for the scientific community, namely, that calorie deprivation during adolescence causes in the short term a lean phenotype and increased insulin sensitivity (in both male and female rats), whereas in the long term it causes an obese phenotype and high peripheral insulin sensitivity in both male and female rats, while in male rats, it causes impaired iBAT redox status (reduced SOD and CAT activity and increased GSH levels).

## 1. Introduction

Undernutrition is still a social problem underlying several kinds of diseases and is a burden for millions of people worldwide. It makes children in particular much more vulnerable to disease and death. In fact, nearly half of the deaths among children under 5 years of age, mostly in low- and middle-income countries, are linked to undernutrition [1]. In terms of the developmental origins of health and disease (DOHaD) paradigm, different cardiometabolic impairments, such as obesity, hypertension and type 2 diabetes mellitus (T2DM), have been associated with protein and/or calorie-energy malnutrition during critical stages of development [2,3,4].

Using a poor-protein diet offered to male rats during the development stage from 30 to 60 days of age, our team provided the first evidence that adolescence is a critical window for programming long-term metabolic disruptions, including inducing a high risk for obesity and T2DM [5]. In our studies, using this rat model, we found that malnutrition during adolescence causes neuroendocrine dysfunction in the hypothalamic–pituitary–adrenal (HPA) and gonadal (HPG) axes and in pancreatic islets [6], and we showed that the effects of undernutrition on metabolism and body composition are more prominent when this nutritional insult occurs during adolescence [7,8]. Similarly, others recently reported emotional disorders and behavior-associated neuronal disruptions as long-term consequences in rats that underwent global food restriction during adolescence [9,10].

Undernutrition is directly involved in unbalanced redox status [11], which has been shown to be associated with different dysfunctions and affects different organs and/or tissues in both rodents and humans. For example, the cognitive function of mice whose mothers experienced protein malnutrition during pregnancy and lactation is impaired [12]. During a period of undernutrition, several changes take place in the body to allow adaptation to the present condition, for example, the activation of transcription factors [13] that are involved in the production of oxidative stress enzymes such as superoxide dismutase (SOD) to remove free radicals [14], which can have long-term consequences for redox status and are associated with energy and glucose metabolism. The balance of redox status in body systems is pivotal for health and is maintained by the balance between several antioxidant systems and the production of reactive oxygen and/or nitrogenous species; when the redox balance is disrupted, biological systems suffer negative effects in different signaling pathways and body functions [15]. In this regard, in the present study, we hypothesized that undernutrition during adolescence would negatively affect body composition and the balance of redox status, affecting glucose homeostasis in rats as a long-term consequence.

Herein, we aimed to assess the short- and long-term effects of undernutrition during adolescence on glucose homeostasis and redox status in metabolic and energy-controlling tissues (liver and interscapular brown adipose tissue (iBAT)) in both male and female rats in view of sex-dependent effects.

## 2. Materials and Methods

### 2.1. Ethical Approval

The protocols were approved by the Ethics Committee for Animal Use and Experiments of the Federal University of Mato Grosso (CEUA/UFMT; process number 23108.021234-2024-73), which adheres to the Brazilian Federal Law number 11.794/2008. Our study complies with the animal ethics checklist as described in the ARRIVE guidelines 2.0 [16].

### 2.2. Experimental Design and Diet Treatment

All the rats were maintained under controlled temperature (23 ± 2 °C), humidity (55 ± 5%) and lighting (12 h light cycle with light on: 06:00 a.m.–06:00 p.m.) conditions throughout the protocol.

Thirty-day-old male and female Wistar rats were randomly assigned to two different groups. The control male (CONT-M, *n* = 32 rats) and female (CONT-F *n* = 32 rats) rats were fed a rodent chow (Nuvital^®^, Curitiba, PR, Brazil) ad libitum throughout the experimental protocol, whereas the undernourished group (FR50-M, *n* = 32 male rats and FR50-F, *n* = 32 female rats) was fed 50% of the amount consumed by the control male and female rats at the same age (from 30 to 60 days old). In each experimental group, half of the rats (*n* = 16) were euthanized at 60 days of age to evaluate the short-term effects of undernutrition. The remaining half (*n* = 16) were maintained until 120 days of age with rodent chow (Nuvital^®^, Curitiba, PR, Brazil) available ad libitum from days 60 to 120 to assess the long-term consequences of adolescent undernutrition. The sample size of 16 was chosen based on previous studies that used rat offspring from at least three to four different litters [17].

This food restriction model was adapted from a well-known malnutrition model used during key developmental stages [18].

### 2.3. Assessment of Food Intake, Drinking Water and Body Weight

To minimize food spillage and water wastage, food and drinking water intakes, as well as body weight, were assessed every 2 days from weaning (22 days old) until the end of the experimental protocol (120 days old). Absolute food intake was calculated as the difference between the total food placed two days before (Food_initial_) and the amount of food remaining (Food_final_), divided by the number of days and the number of rats per cage: [FI_(g)_ = (Food_initial_−Food_final_)/2/4]. A similar calculation was performed to assess drinking water intake.

Relative food intake and drinking water intake were calculated as the value of absolute food (in grams) or absolute drinking water (in milliliters) intake divided by the mean body weight of the 4 rats in the same cage.

### 2.4. Glucose–Insulin Homeostasis Assessment

An intraperitoneal glucose tolerance test (ipGTT) was performed after food deprivation for 12 h (6:00 a.m.–6:00 p.m.), with free access to drinking water. Blood samples were initially collected before the initial glucose load (0 min, basal glycemia), after which a glucose load (2 g/kg body weight) was injected intraperitoneally into the conscious rats. After that, at 30, 60, 90 and 120 min, blood samples were obtained from the tail vein, and the glucose concentration was determined by a digital glucometer (Accu-Chek^®^ Performa, Roche, Basel, Switzerland), as previously reported [19].

An intraperitoneal insulin tolerance test (ipITT) was performed on the same rats that underwent an ipGTT. Thus, 48 h after the ipGTT, the rats were fasted for 4 h (2:00 a.m.–6:00 p.m.), with free access to drinking water. Blood samples for blood glucose measurements were collected immediately before insulin injection (0 min, basal), after which insulin (1 IU/kg bw) was administered intraperitoneally to conscious rats. After that, at 15, 30, 45 and 60 min after insulin injection, blood samples were obtained from the tail vein, and the glucose concentration was determined by a digital glucometer (Accu-Chek^®^ Performa, Roche), as previously reported [19].

Thereafter, the rate of glucose tissue uptake or the rate constant for plasma glucose disappearance (Kitt) was calculated by the formula 0.693/(t_1/2_). The plasma glucose half-life was calculated from the slope of the least-square analysis of the plasma glucose concentrations during the linear phase of decline [20].

### 2.5. Assessment of Body Mass Composition

To assess the short- and long-term consequences of undernutrition during adolescence, half of the rats (*n* = 16) in each group were euthanized at 60 days of age, and the other half (*n* = 16) were euthanized at 120 days of age.

The overnight-fasted rats were euthanized by decapitation. Next, blood, liver, iBAT and white adipose visceral tissue (periovarian or periepididimal, retroperitoneal and mesenteric), as well as skeletal muscle (soleus and extensor digitorum longus, EDL) as a representative of lean mass, were removed and weighed.

The adiposity index was used to assess the amount of body fat of the rats. For this, the sum of the visceral fat deposits normalized to the body weight of the rats was used as follows: adiposity index = [(periovarian or periepididimal + retroperitoneal + mesenteric fat)/body weight × 100]. Similarly, the skeletal lean mass index was calculated as the sum of the values normalized by the rat body weight: lean mass index = [(soleus + EDL)/body weight × 100], as previously described as the method to infer adiposity and lean mass index [17].

### 2.6. Assessment of the Oxidative Status of the Liver and iBAT

To investigate the redox status in the liver and iBAT, the enzymatic antioxidant activity of catalase (CAT) and SOD, as well as the concentration of the nonenzymatic antioxidant reduced glutathione (GSH), was evaluated according to the methods described in a previous publication [21].

### 2.7. Statistical Analyses

The results are given as the mean ± SD and were subjected to the Shapiro-Wilk normality test. Data that were found to have a Gaussian distribution were subjected to a parametric test (one-way analysis of variance (one-way ANOVA), followed by Tukey’s multiple comparisons posttest). For data that did not follow a Gaussian distribution, a nonparametric test was used (Kruskal-Wallis’ test followed by Dunn’s multiple comparisons post hoc test).

Tests were performed using GraphPad Prism version 8.0 for Windows (GraphPad Software Inc., San Diego, CA, USA), in which *p* < 0.05 was considered to indicate statistical significance.

## 3. Results

### 3.1. Food and Drinking Water Intake and Body Weight Gain

**Figure 1:**
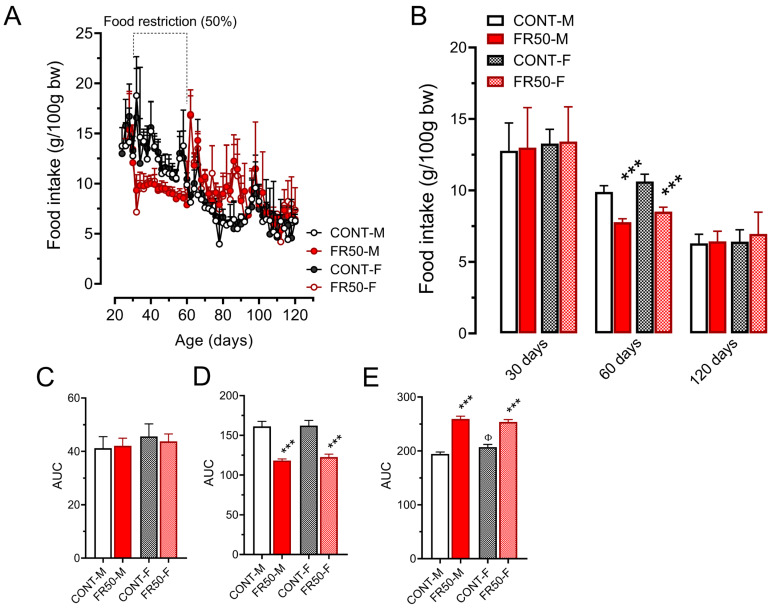
Food intake throughout the experimental period (**A**) and at 30, 60 and 120 days of age (**B**). The data are presented as the means ± SDs (*n* = 4 litters). The area under the curve (AUC) for each period of dietary treatment: (**C**). before undernutrition, from 22 to 30 days old; (**D**). during undernutrition, 30 to 60 days old; and (**E**). after undernutrition, from 60 to 120 days old. The symbol *** *p* < 0.001 indicates a comparison between CONT and FR50, and ^Φ^ *p* < 0.05 indicates a comparison between males (M) and females (F) using one-way ANOVA followed by Tukey’s post hoc test. Data from (**B**,**C**) were analyzed using the Kruskal-Wallis’ test with Dunn’s multiple comparison post hoc test. CONT-M, control group of male rats; CONT-F, control group of female rats; FR50-M, undernourished group of male rats; FR50-F, undernourished group of female rats.

Food intake did not differ among the groups before undernutrition (*p* > 0.05; Figure 1A–C). In terms of food intake throughout the period of undernutrition (from 30 to 60 days of age), as expected, the area under the curve (AUC) of FR50-M rats was 26.66% lower than the AUC of CONT-M rats, and the area under the curve of FR50-F rats was 24.40% lower than that of CONT-F rats (*p* < 0.001; Figure 1A–D). At 60 days of age, compared with CONT-M rats, FR50-M rats consumed 20.37% less, and compared with CONT-F rats, FR50-F rats consumed 18.45% less (*p* < 0.001; Figure 1A,B). However, food intake did not differ between CONT-M and CONT-F or between FR50-M and FR50-F at 60 days of age or throughout the period of undernutrition (*p* > 0.05; Figure 1A,B,D).

During the postundernutrition period (from 60 to 120 days of age), food intake increased by 33.35% in the FR50-M group compared with that in the CONT-M group and by 23.88% in the FR50-F group compared with that in the CONT-F group (*p* < 0.001; Figure 1A,E). In addition, the food intake of the CONT-F group was 5.30% greater than that of the CONT-M group (*p* < 0.05; Figure 1A,E), but it did not differ between the FR50-M group and the FR50-F group (*p* > 0.05; Figure 1A,E). At 120 days of age, there were no significant differences among the groups (*p* > 0.05; Figure 1A,B,D).

**Figure 2:**
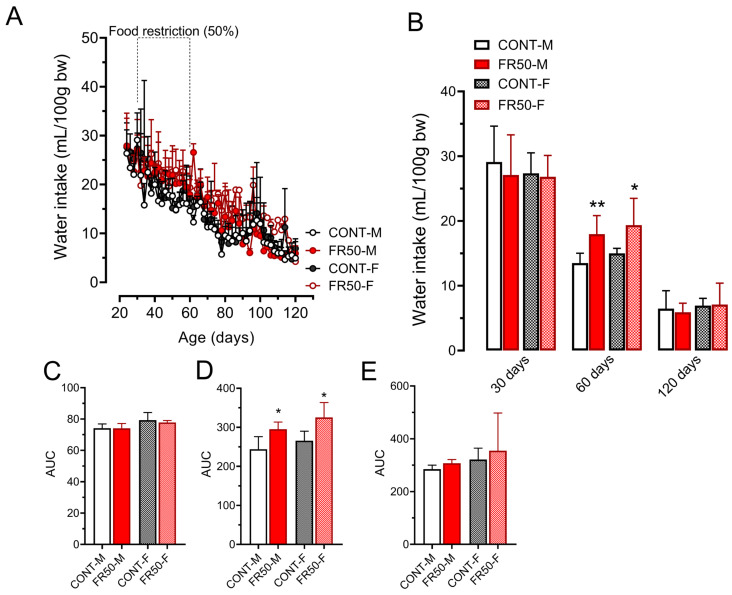
Drinking water intake throughout the experimental period (**A**) and at 30, 60 and 120 days of age (**B**). The data are presented as the means ± SDs (*n* = 4 litters). The area under the curve (AUC) for each period of dietary treatment: (**C**). before undernutrition, from 22 to 30 days old; (**D**). during undernutrition, 30 to 60 days old; and (**E**). after undernutrition, from 60 to 120 days old. * *p* < 0.05 and ** *p* < 0.01 indicate comparisons between CONT and FR50 using one-way ANOVA followed by Tukey’s post hoc test. The data in Panels (**B**,**E**) were analyzed using the Kruskal-Wallis’ test, followed by Dunn’s multiple comparison test. CONT-M, control group of male rats; CONT-F, control group of female rats; FR50-M, undernourished group of male rats; FR50-F, undernourished group of female rats.

The amount of drinking water did not differ between the groups before (*p* > 0.05; Figure 2A–C) or after the undernutrition treatment (*p* > 0.05; Figure 2A,B,E). On the other hand, during the period of undernutrition, compared with CONT-M, the intake of drinking water increased by 21.01% in the FR50-M group (*p* < 0.05; Figure 2A,D) and by 22.47% in the FR50-F group vs. the CONT-F group (*p* < 0.05; Figure 2A,D). At 60 days of age, compared with CONT-M rats, FR50-M rats drank 33.61% more water (*p* < 0.01; Figure 2A,B), and compared with CONT-F rats, FR50-F rats drank 29.13% more water (*p* < 0.01; Figure 2A,B). Water intake did not differ between CONT-M and CONT-F or between FR50-M and FR50-F at 60 days of age or throughout the period of undernutrition (*p* > 0.05; Figure 2A,B,D).

**Figure 3:**
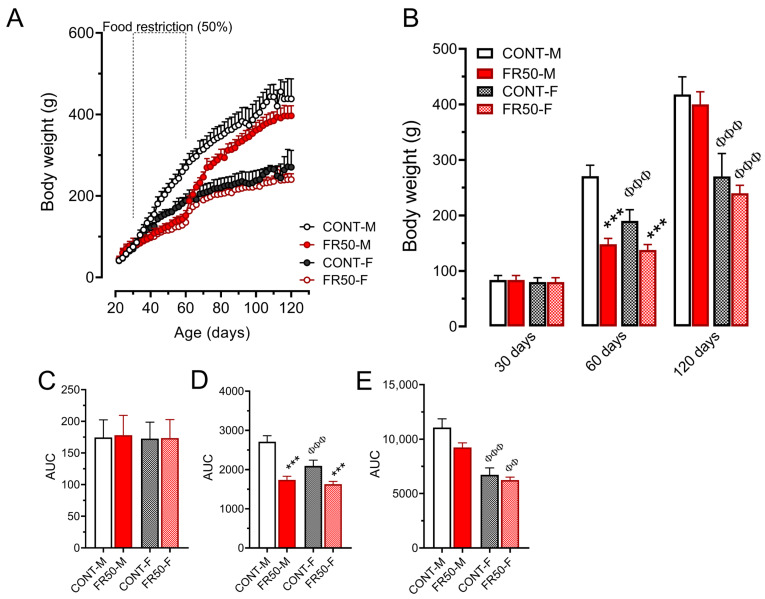
Body weight gain throughout the experimental period (**A**) and at 30, 60 and 120 days of age (**B**). The data are presented as the means ± SDs (*n* = 16 rats). The area under the curve (AUC) for each period of dietary treatment: (**C**). before undernutrition, from 22 to 30 days old; (**D**). during undernutrition, 30 to 60 days old; and (**E**). after undernutrition, from 60 to 120 days old. The symbol *** *p* < 0.001 indicates a comparison between CONT and FR50, and ^ΦΦ^ *p* < 0.01 and ^ΦΦΦ^ *p* < 0.001 indicate a comparison between males (M) and females (F) using one-way ANOVA followed by Tukey’s post hoc test. The data in Figure (**B**,**C**,**E**) were analyzed using the Kruskal-Wallis’ test followed by Dunn’s multiple comparison test. CONT-M, control group of male rats; CONT-F, control group of female rats; FR50-M, undernourished group of male rats; FR50-F, undernourished group of female rats.

The body weights of the rats did not differ among the groups before the period of undernutrition (*p* > 0.05; Figure 3A–C). On the other hand, in terms of body weight gain throughout the period from 30 to 60 days of age, the body weight of the FR50-M rats was 36.37% lower than that of the CONT-M rats, and the body weight of the FR50-F rats was 22.74% lower than that of the CONT-F rats (*p* < 0.001; Figure 3A,D). While the AUC of the CONT-F group was 22.70% smaller than that of the CONT-M group (*p* < 0.001; Figure 3D), it did not differ between FR50-F and FR50-M (*p* > 0.05; Figure 3D).

At 60 days of age, in both sexes, compared with CONT rats, FR50 rats were leaner. In relation to the CONT-M group, the body weight of the FR50-M group was reduced by 45.27% (*p* < 0.001; Figure 3A,B), the body length was reduced by 13.18% (*p* < 0.001; CONT-M: 19.65 ± 0.79 vs. FR50-M: 17.06 ± 1.25), and the body weight of the FR50-F group was 27.57% lower than that of the CONT-F group (*p* < 0.001; Figure 3A,B), and the body length was reduced by 11.86% (*p* < 0.01; CONT-F: 18.05 ± 0.19 vs. FR50-F: 15.91 ± 1.18). While the body weight of CONT-F was 29.67% lower than that of CONT-M (*p* < 0.001; Figure 3A,B), it did not differ between FR50-M and FR50-F (*p* > 0.10; Figure 3A,B). The body length was 8.14% shorter in CONT-F rats than in CONT-M rats (*p* < 0.05; CONT-M: 19.65 ± 0.79; CONT-F: 18.05 ± 0.19) and 6.74% shorter in FR50-F rats than in FR50-M rats (*p* = 0.315; FR50-M: 17.06 ± 1.25; FR50-F: 15.91 ± 1.18).

The body weights of the FR50-M and FR50-F rats did not differ from those of their respective counterparts during the postundernutrition period (*p* > 0.05; Figure 3A,E) nor at 120 days of age (*p* > 0.05; Figure 3A,B). However, compared with the CONT-M group, the CONT-F group was lighter (at 120 days: 35.28%, *p* < 0.001; Figure 3A,B; and from 60 to 120 days: 37.03%, *p* < 0.001; Figure 3E). Similarly, at 120 days of age, the body weight of the FR50-F rats was reduced by 39.95% in relation to that of the FR50-M rats (*p* < 0.001; Figure 3A,B) and was reduced by 33.40% at 60 days of age (*p* < 0.01; Figure 3E).

### 3.2. Murinometric Parameters

**Figure 4:**
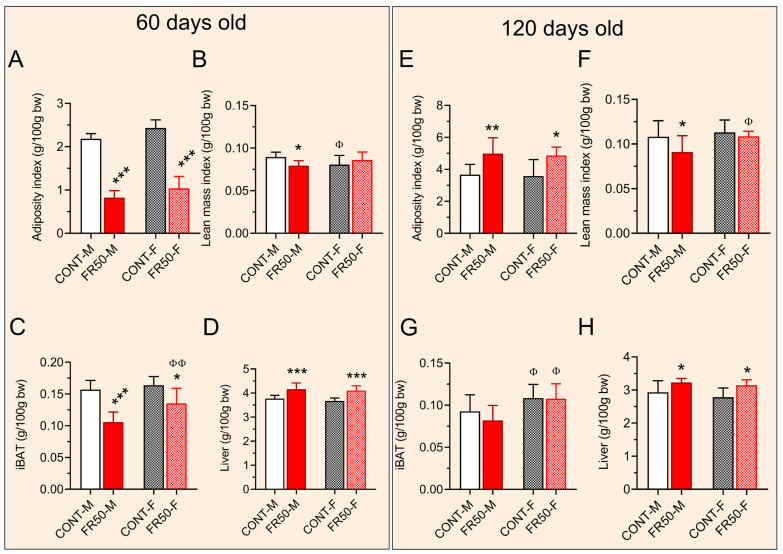
Short- and long-term effects of pubertal undernutrition on body composition. The data are presented as the means ± SDs (*n* = 16 rats). (**A**–**D**). The short-term effects and (**E**–**H**) the long-term effects. Symbols * *p* < 0.05, ** *p* < 0.01, *** *p* < 0.001 depict comparisons between CONT and FR50, and ^Φ^ *p* < 0.05 and ^ΦΦ^ *p* < 0.01 depict comparisons between males (M) and females (F) using one-way ANOVA followed by Tukey’s post hoc test. CONT-M, control group of male rats; CONT-F, control group of female rats; FR50-M, undernourished group of male rats; FR50-F, undernourished group of female rats.

At 60 days of age, the adiposity index was reduced by 62.19% in the FR50-M group and by 57.34% in the FR50-F group (*p* < 0.001; Figure 4A). There was no significant difference between CONT-M and CONT-F or between FR50-M and FR50-F (*p* > 0.05; Figure 4A). Compared with that in CONT-M, the lean mass index in FR50-M was reduced by 11.27% (*p* < 0.05; Figure 4B) but it was not significantly different between FR50-F and CONT-F (*p* > 0.05; Figure 4B). Compared to CONT-M rats, the lean mass index in CONT-F rats was decreased by 9.94% (*p* < 0.05; Figure 4B) but it did not differ between FR50-F and FR50-M rats (*p* > 0.05; Figure 4B).

At 60 days of age, compared with CONT-M rats, FR50-M rats displayed 32.70% lower iBAT (*p* < 0.001; Figure 4C). Similarly, the iBAT in the FR50-F group was reduced by 17.50% compared with that in the CONT-F group (*p* < 0.05; Figure 4C). While iBAT did not differ between CONT-F and CONT-M, it increased by 28.04% in FR50-F compared to FR50-M (*p* < 0.01; Figure 4C). Compared with the controls, the liver mass at 60 days of age increased by 10.45% in the FR50-M group and by 12.79% in the FR50-F group (*p* < 0.001; Figure 4D). There was no significant difference in liver mass between CONT-F and CONT-M or between FR50-F and FR50-M (*p* > 0.05; Figure 4D).

At 120 days of age, the adiposity index increased by 36% in FR50-M rats compared to CONT-M rats (*p* < 0.01; Figure 4E) and in FR50-F rats compared to CONT-F rats (*p* < 0.05; Figure 4E). There was no significant difference between CONT-M and CONT-F or between FR50-M and FR50-F (*p* > 0.05; Figure 4E). Compared with that in CONT-M rats, the lean mass index in FR50-M rats was reduced by 15.65% (*p* < 0.05; Figure 4F), but it was not significantly different between FR50-F and CONT-F rats (*p* > 0.05; Figure 4F). A comparison of the CONT-F and CONT-M rats revealed that the lean mass index did not differ between them, but when the FR50-F and FR50-M rats were compared, it increased by 19.32% in the FR50-F group (*p* < 0.05; Figure 4F).

At 120 days of age, the iBAT mass did not differ between the FR50-M and CONT-M rats or between the FR50-F and CONT-F rats (*p* > 0.05; Figure 4G); however, the iBAT mass increased in both sexes in both dietary-treated groups, where the iBAT of CONT-F was 16.4% greater than that of CONT-M and 31.34% greater in FR50-F than in FR50-M (*p* < 0.05; Figure 4G). Compared with the controls, the liver mass at 120 days of age increased by 10.34% in the FR50-M group and by 12.97% in the FR50-F group (*p* < 0.05; Figure 4H). There was no significant difference in liver mass between CONT-F and CONT-M or between FR50-F and FR50-M (*p* > 0.05; Figure 4H).

### 3.3. Glucose–Insulin Homeostasis

**Figure 5:**
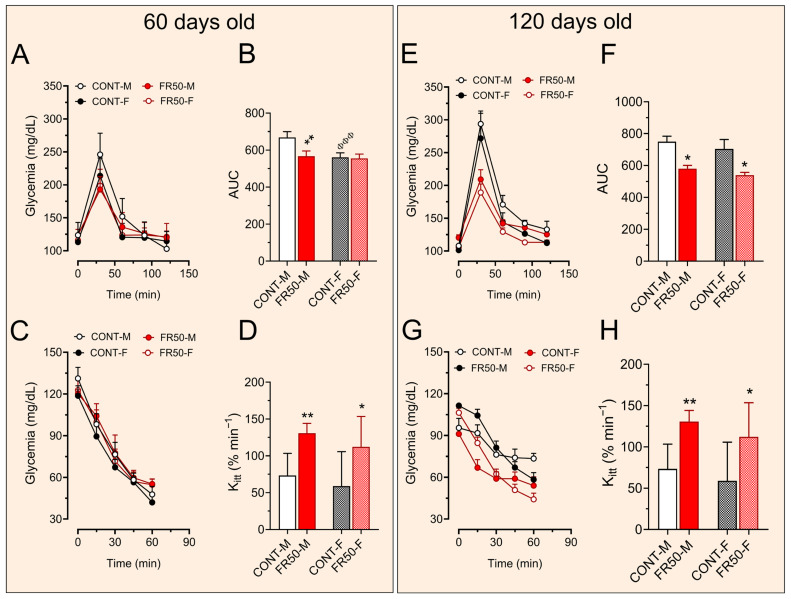
Short- and long-term effects of pubertal undernutrition on glucose–insulinemia homeostasis. The data are presented as the means ± SDs (*n* = 8 rats). The area under the curve (AUC) of the ipGTT (**B**) for rats at 60 days and (**F**) for rats at 120 days old, and the short-term effect (**A**–**D**) and the long-term effect (**E**–**H**). Symbols * *p* < 0.05, ** *p* < 0.01 depict comparisons between CONT and FR50, and ^ΦΦΦ^ *p* < 0.001 depicts comparisons between males (M) and females (F) using one-way ANOVA followed by Tukey’s post hoc test. CONT-M, control group of male rats; CONT-F, control group of female rats; FR50-M, undernourished group of male rats; FR50-F, undernourished group of female rats.

At 60 days of age, the fasting glycemia of the rats did not differ among the groups (*p* > 0.05, CONT-M: 123.8 ± 19.24 vs. FR50-M: 123.8 ± 8.73 and CONT-F: 113.3 ± 10.11 vs. FR50-F: 116.01 ± 6.98). During the ipGTT, the AUC of glycemia in the FR50-M rats was reduced by 15.17% compared with that in the CONT-M rats (*p* < 0.01; Figure 5A,B), even though it was not significantly different between the FR50-F and CONT-F rats (*p* > 0.05; Figure 5A,B). Additionally, the AUCs of CONT-F and CONT-M did not differ (*p* > 0.05; Figure 5A,B). Compared with that in CONT-M rats, the amount of Kitt at 60 days of age increased by 78.40% (*p* < 0.01; Figure 5C,D), and compared to CONT-F rats, it increased by 90.59% in FR50-F (*p* < 0.05; Figure 5C,D). Comparisons between the CONT-F and CONT-M groups and between the FR50-F and FR50-M groups did not reveal significant differences (*p* > 0.05; Figure 5C,D).

At 120 days of age, the fasting glycemia of the FR50-M group was 11.48% greater than that of the CONT-M group (*p* < 0.05, CONT-M: 108.01 ± 4.08 vs. FR50-M: 120.4 ± 9.63), but it did not differ between the FR50-F group and the CONT-F group (*p* > 0.05, CONT-F: 101.4 ± 3.34 vs. FR50-F: 105.5 ± 4.14). While fasting glycemia did not differ between CONT-F and CONT-M rats (*p* > 0.05), it was 12.38% lower in FR50-F rats than in FR50-M rats (*p* < 0.01).

At 120 days of age, the AUC of glycemia in the FR50-M rats remained lower than that in the CONT-M rats (22.58%, *p* < 0.05; Figure 5E,F) and was 23.39% lower in the FR50-F rats than in the CONT-F rats (*p* < 0.05; Figure 5E,F). The AUCs of glycemia during the ipGTT were not significantly different between CONT-F and CONT-M rats or between FR50-F and FR50-M rats (*p* > 0.05; Figure 5E,F). Compared with that in the CONT-M group, the amount of Kitt in the FR50-M group was 225% greater (*p* < 0.01; Figure 5G,H), and it was 166.44% greater in the FR50-F group than in the CONT-F group (*p* < 0.05; Figure 5G,H). Comparisons between the CONT-F and CONT-M groups and between the FR50-F and FR50-M groups did not reveal significant differences (*p* > 0.05; Figure 5G,H).

### 3.4. Assessment of Liver and iBAT Redox Status

**Figure 6:**
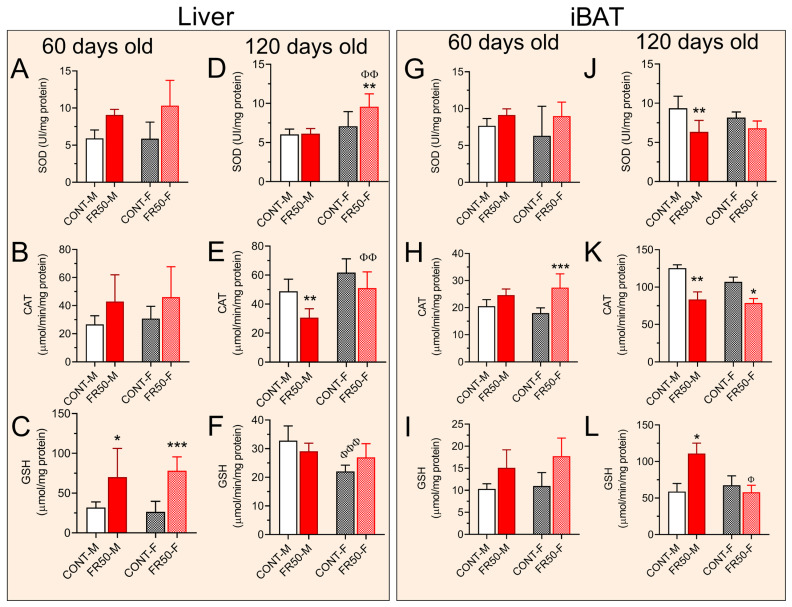
Short-and long-term effects of pubertal undernutrition on the liver and iBAT redox status. The data are presented as the means ± SDs (*n* = 8 rats). The short-term effects (**A**–**C**) on liver and (**G**–**I**) on iBAT, and the long-term effects (**D**–**F**) on liver and (**J**–**L**) on iBAT. Symbols * *p* < 0.05, ** *p* < 0.01, *** *p* < 0.001 depict comparisons between CONT and FR50, and ^Φ^ *p* < 0.05, ^ΦΦ^ *p* < 0.01, and ^ΦΦΦ^ *p* < 0.001 depict comparisons between males (M) and females (F) using one-way ANOVA followed by Tukey’s post hoc test. CONT-M, control group of male rats; CONT-F, control group of female rats; FR50-M, undernourished group of male rats; FR50-F, undernourished group of female rats.

At 60 days of age, the redox statuses of the SOD and CAT biomarkers in the liver (*p* > 0.05; Figure 6A,B), as well as the SOD activity and GSH levels in the iBAT (*p* > 0.05; Figure 6G,I), did not differ among the groups or between the sexes.

At the same age, compared with those in the CONT-M group, the liver GSH levels in the FR50-M group increased by 121.14% (*p* < 0.05; Figure 6C), and compared with those in the CONT-F group, they increased by 195.88% in the FR50-F group (*p* < 0.001; Figure 6C). The liver GSH levels did not differ between CONT-F and CONT-M or between FR50-F and FR50-M (*p* > 0.05; Figure 6C). In turn, the activity of CAT in the iBAT of FR50-M did not differ from those in CONT-M (*p* > 0.05; Figure 6H) but increased by 52.41% in FR50-F in comparison with those in CONT-F (*p* < 0.001; Figure 6H). The activity of CAT in the iBAT from CONT-F vs. CONT-M and from FR50-F vs. FR50-M were not significantly different (*p* > 0.05; Figure 6H).

At 120 days of age, the activity of SOD in the liver did not differ among the groups (*p* > 0.05; Figure 6D). Compared with those in CONT-M, the CAT activity in the liver were reduced by 37.30% in FR50-M (*p* < 0.01; Figure 6E), while there was no significant difference between FR50-F and CONT-F (*p* > 0.05; Figure 6E) or between CONT-F and CONT-M (*p* > 0.05; Figure 6E). Compared with those in the livers of the FR50-M rats, the activity of CAT in the livers of the FR50-F rats were increased by 66.70% (*p* < 0.01; Figure 6E). While we did not observe a significant difference in the levels of GSH in the liver between FR50-M and CONT-M, FR50-F and CONT-F, or FR50-F and FR50-M (*p* > 0.5; Figure 6F), compared with those in the CONT-M group, the liver GSH levels in the CONT-F group were reduced by 32.73 (*p* < 0.001; Figure 6F).

At 120 days of age, the activity of SOD in the iBAT of FR50-M rats was reduced by 32.22% compared with that in the iBAT of CONT-M rats (*p* < 0.01; Figure 6J), but the activity did not differ between FR50-F and CONT-F, CONT-F and CONT-M, or FR50-F and FR50-F (*p* > 0.05; Figure 6J). The iBAT CAT activity was reduced by 33.36% in the FR50-M group compared with that in the CONT-M group (*p* < 0.01; Figure 6K) and by 26.50% in the FR50-F group compared with that in the CONT-F group (*p* < 0.05; Figure 6K). There was no significant difference between CONT-M and CONT-F or between FR50-F and FR50-M (*p* > 0.05; Figure 6K).

The GSH levels of the iBAT at 120 days of age increased by 88.53% in FR50-M rats compared with those in CONT-M rats (*p* < 0.05; Figure 6L), whereas significant differences were not detected between FR50-F and CONT-F rats or between CONT-F and CONT-M rats (*p* > 0.05; Figure 6L). The level of GSH in the iBAT of the FR50-F rats was 47.79% lower than that in the iBAT of the FR50-M rats (*p* < 0.05; Figure 6L).

## 4. Discussion

In the current study, we demonstrate that undernutrition during adolescence affects body composition, leading to high food intake and associated rapid catch-up growth in male and female rats throughout the period of nutritional recovery. In addition, the short-term effect of the lean phenotype is not long-term, possibly indicating that these rats are at high risk for developing obesity later in life.

With respect to the increased adiposity index observed as a long-term consequence in rats that were undernourished during adolescence, these animals did not ingest adequate amounts of nutrients, leading to weight loss during undernutrition. It is suggested that chronic hunger must have triggered a stimulus that may have modulated endocrine changes [13,14] in these rats, leading to the intake of more food and increasing body weight throughout life; even a slight increase can gradually contribute to fat accumulation, especially if it is associated with high insulin sensitivity. Herein, we did not quantify insulin secretion to assess pancreatic islet function or insulin signaling pathways in the liver to better explore the possibility of lipid anabolism, which is one of the limitations of the current study.

During fasting, as well as during chronic hunger conditions, as in the present study, the level of ghrelin increases and that of leptin decreases, which increases appetite by stimulating hypothalamic NPY/AgRP neurons and increasing the secretion of growth hormone (GH), which acts on hepatic tissue. With respect to hunger conditions, hepatic tissue develops resistance to avoid insulin growth factor type 1 (IGF-1) hypersecretion and its overstimulation in peripheral tissues [22]. During the period of nutritional recovery, when the animals were once again given ad libitum food, as expected, greater food intake was observed in both sexes, since at this stage, the metabolism of the rats that were subjected to malnutrition had altered because of the period of malnutrition. Thus, they tended to eat more and consequently gain more weight, which is a physiological effect of trying to overcome caloric deficiency [23]. However, when malnutrition affects transcription factors, this can lead to delayed recovery, which can contribute to the development of obesity, among other diseases, in the long term, which may be associated with neuropeptide derangement (such as a reduction in NPY) in rats [10].

During a chronic starvation period, there is reduced phosphorylation and acetylation of signal transducer and activator of transcription (STAT) [24], increased production of fibroblast growth factor 21 (FGF-21) and decreased production of IGF-1 [25,26], as well as increased protein sirtuin 1 (SIRT-1) activity, leading to deacetylation and inactivation of STAT3 and deacetylation and degradation of sterol regulatory element binding protein-1 (SREBP-1), reducing lipogenesis and cholesterol synthesis [27]. In addition, the evidence suggests that, similar to SIRT-1, under calorie restriction, SIRT-3 plays a relevant role in regulating redox balance by deacetylating isocitrate dehydrogenase 2 (IDH2), which increases the level of the reducing equivalent NADPH [28] that is used by glutathione reductase to convert oxidized glutathione (GSSG) to GSH, a cofactor for glutathione peroxidase (GPx) that protects against reactive oxygen species [29], which may explain the high liver GSH in FR50 rats at 60 days of age, suggesting that metabolic adaptation is a direct effect of nutrient scarcity in undernutrition. Although reduced blood and erythrocyte GSH levels have been linked to increased plasma biomarkers of oxidative damage in severe childhood undernutrition [30], understanding how shifts in oxidative balance contribute to metabolic dysfunction is essential and deserves attention in further studies that link it to famine conditions.

During the period of free food supply, immediately after malnutrition, catch-up growth results from two apparently conflicting actions associated with the action of GH and ghrelin [22], since in this period, hepatic resistance to GH ends, and the elevated GH levels result in elevated IGF-1 levels that act on target tissues to promote higher than normal protein synthesis and cell division, leading to catch-up growth. Leptin production gradually increases as adipose stores increase, which leads to greater inhibition of central GH production. Moreover, ghrelin secretion decreases and further decreases GH secretion [22]. Given that adolescence is pivotal for life development, in which a growing body displays high metabolic demands [31], changes in the availability of nutrients, hormones and metabolic signals must contribute to the long-term consequences of metabolic derangement in late life.

Data derived from clinical studies related to obesity and oxidative stress have established a correlation between oxidative stress biomarkers and high body mass indices [32,33]. Unlike what was seen in male rats subjected to protein malnutrition during adolescence [5,6], in the present study, glucose intolerance and insulin resistance were not observed. As has already been shown in the literature, malnutrition is defined as an inadequate intake of nutrients such as proteins, vitamins, and minerals [34], resulting in the body’s inability to meet the demand necessary for growth, development, and maintenance of specific functions in a physiological way, thus increasing the predisposition to the appearance and/or progression of metabolic diseases [34] and inducing diverse effects throughout life [35].

Our results show that the animals that were malnourished during adolescence presented a greater tolerance to glucose, as well as a greater peripheral sensitivity to insulin, in both sexes, although the males had fasting hyperglycemia at 120 days of age. These findings suggest that the peripheral sensitivity of malnourished rats to insulin is high, suggesting that fast-acting counterregulatory hormones (glucagon and/or adrenaline) participate in the control of glucose homeostasis in this animal model. Studies have shown that mice treated for 8 weeks with a low-protein diet present greater production of hepatic glycogen, which is associated with increased hepatic sensitivity to insulin [36]. In our study, we did not assess insulin signaling pathways in the liver, which is one of the limitations in the body of data presented herein. In addition, studying the mechanisms underlying high peripheral sensitivity to insulin in experimental models of metabolic dysfunction due to undernutrition is needed. As we previously reported, rats that are undernourished during lactation display high peripheral insulin sensitivity, which has been linked to impairment of glucocorticoid signaling in iBAT [37] and action on pancreatic islets and insulin secretion [38].

The results obtained in the present study show that malnutrition was able to modulate the antioxidant system in iBAT without causing major effects on the liver in the long term. Our data revealed a reduction in the activity of SOD in FR50 male but not in female rats and an increase in GSH only in FR50 male rats. In addition, the magnitude of the reduction in the activity of CAT in the iBAT of the FR50 male rats was greater than that of the female rats, suggesting that oxidative damage to the thermogenic system of male rats is a long-term consequence, whereas in females, a certain degree of resilience to the effects of undernutrition upon metabolism can be detected. An imbalance between the formation and removal of reactive oxygen species in the body, resulting from a decrease in endogenous antioxidants or an increase in the generation of oxidant species, generates a pro-oxidant state that favors the occurrence of oxidative damage to macromolecules and cellular structures, which can result in cell death and dysfunction [39,40].

Among antioxidant biomarkers, SOD is the first detoxification enzyme and the most powerful antioxidant in cells [41], and its decrease may be associated with excessive production of superoxide anions, which results in the formation of other reactive species, including peroxynitrite and the hydroxyl radical, which are potent inducers of damage to DNA, proteins, and lipids [42]. Reductions in SOD and CAT have been associated with obesity and metabolic dysfunction [43], which may suggest that, compared with female rats, FR50-treated male rats are more prone to develop metabolic derangement and obesity. On the other hand, since no reduction in these markers was observed in FR50 female rats as a long-term consequence, it can be inferred that the females that were malnourished developed adaptation to oxidative stress and recovered their function. This adaptation might be caused by estrogen, which acts directly as an antioxidant against free radicals [44].

Recent reports indicate that estrogen plays a role in protecting cells from oxidative stress [45] and may also contribute to cardiovascular health [46] and metabolic functions such as obesity and diabetes prevention [46,47]. Herein, the lack of measurements of sex hormones (estrogen and testosterone) represents another limitation of the study.

In terms of short- and long-term effects, hepatomegaly was observed in both sexes, which may be associated with the programming of greater biochemical functionality of the liver due to the effects of metabolic stress during malnutrition [48,49].

In terms of the short-term effect of malnutrition on iBAT, a reduction in the weight of this tissue was observed. Notably, iBAT is important for the thermogenic response and energy balance in small mammals, with the main function of oxidizing lipids to produce heat, and is therefore specialized in adaptive thermogenesis [50]. Diet can also activate iBAT in a similar manner (diet-induced thermogenesis); this activity influences food behavior and energy balance. In this context, transgenic mice lacking brown adipose tissue develop obesity [51].

Several studies in animal models have shown that obesity is related to reduced activity of iBAT. Among these models are those with genetic alterations, such as fa/fa mice [52,53], db/db mice [54,55] and animals with monosodium-glutamate-induced obesity [56,57].

Given the low weight of the soleus muscle in males, malnutrition in adolescence may act as an imbalance factor, causing changes in the tissues and structures of the organs. Skeletal muscle tissue is sensitive to protein malnutrition because it is a protein reservoir in the body. Therefore, when protein deficiency occurs in the diet, this tissue becomes a target for depletion [58]. The results of the present study corroborate those of a previous study in which protein malnutrition reduced the weight of muscle tissue [59], which may have occurred because of the loss of tissue proteins. In our study, as a long-term effect, no reduction in lean mass was observed in females, but a reduction in lean mass was detected in males. This may be due to the presence of sex hormones (estrogens) in females, which protect the regulation of body weight and energy expenditure.

## 5. Conclusions

Our data support the hypothesis that undernutrition during adolescence programs high accumulation of visceral adiposity and peripheral hypersensitivity to insulin in both males and females. It was also concluded that malnutrition during adolescence was able to induce less redox protection in the thermogenic tissue of male rats. On the other hand, females, regardless of nutritional stress, presented increased defenses against reactive oxygen species. In analyzing the results presented in this work, it is worth mentioning that the way in which metabolism reacts to caloric restriction (chronic hunger) shows that any form of malnutrition should be avoided, not only in adolescents but also in people in general, because when in a critical phase of development, the results of malnutrition can be surprising and devastating.

## Data Availability

The raw data supporting the conclusions of this article will be made available by the authors on request.
